# Supplementary material for: Effectiveness of aerobic physical exercise on depression symptoms in adults: A protocol for developing a systematic review and meta-analysis of randomized clinical trials
Source: PLoS One. 2025 Jun 2;20(6):e0314846. doi: 10.1371/journal.pone.0314846 (PMC12129328; doi:10.1371/journal.pone.0314846)
Supplement: Attachment — Submitted filename: Response to Reviewers -Plos One.docx [file pone.0314846.s002.docx]

**Response to Reviewers**

**Manuscript number:** PONE-D-24-51714

**Title:** Effectiveness of aerobic exercise on depression symptoms in adults: A protocol for a systematic review and meta-analysis of randomized clinical trials

**Dear Reviewers,**

Firstly, I would like to express my gratitude, on behalf of all the authors of the manuscript, to the esteemed reviewers for raising considerations of utmost importance, which are highly relevant to improving the article.

It is important to note the distinguished reviewers that the manuscript submitted to PLOS ONE aims to summarize scientific evidence on the effect of aerobic exercise in the treatment of depression in adults. We have made all the requested adjustments, which have been incorporated into the manuscript itself, with the changes highlighted in red font.

These modifications are also listed below:

| **Reviewer #1** | **Authors' response:** |
| --- | --- |
| Reviewer: Although the protocol is well-structured and follows robust guidelines for systematic reviews and meta-analyses, some aspects could be improved to enhance the clarity and methodological quality of the work. Below are my suggestions for improvement:  -  Publication Bias Assessment: While the use of a random-effects model for the meta-analysis is appropriate, it is unclear how the protocol will address publication bias. It is recommended to include methods for identifying and managing this issue, such as funnel plots or statistical tests like Egger's test. | We appreciate your input and concur with your position. Kindly review the alterations that have been made.  “For the assessment of publication bias, Egger's statistical test will be implemented, a sophisticated tool used to enhance the consistency of funnel plot analysis [14]. In cases where publication bias is identified, a technique that may be used to correct it is the trim-and-fill method, which adjusts the distribution of studies in an asymmetric funnel plot [15]”. |
| - Qualitative Data Analysis: The protocol mentions the analysis of categories and emerging themes (qualitative data) but does not clarify under which circumstances these data will be analyzed or presented. Given the quantitative focus of the study, it would be helpful to specify the role and relevance of these qualitative data within the overall scope of the review. | We appreciate your input and concur with your position, we decided exclude the qualitative data analysis, we agree with you. “Quantitative results will include effect size, measures of variability such as confidence intervals, standard deviation, and p-values, as well as other data relevant for interpreting the results. |
| **Reviewer #2** | |
| The authors intended to develop a protocol for reviewing studies involving aerobic exercise and depression; however, I believe the study promotes something but missed the opportunity to deliver. There are numerous good examples of systematic reviews, so why should this protocol be followed? | We appreciate your input and concur with your position. Kindly review the alterations that have been made. We have revised the rationale for conducting this systematic review based on the existing literature. The main objective was to overcome the limitations identified in previous reviews, enhancing the study's approach and theoretical foundation.  “Therefore, this study is justified by the need to understanding of how different intensities and types of aerobic exercise can influence the reduction of depression levels in adults. Various international guidelines, such as those from the United Kingdom and Australia, recommend physical exercise as part of depression treatment [4,5]. However, these guidelines do not provide clear and consistent recommendations regarding the dose or modality of exercise [6]. In turn, the American Psychiatric Association recommends any dose of aerobic exercise or strength training [7], while the Australian guidelines already suggest a combination of vigorous aerobic and strength exercises at least twice a week [5].Supposedly, a network meta-analysis has shown that dose and modality influence cognition [8]. In this regard, it is essential to consider the need for reviews like this one, given the numerous designs of meta-analyses and systematic reviews that, despite their relevance, have presented methodological limitations preventing fundamental conclusions. Under these conditions, considering the isolated analysis of aerobic exercises may be a promising alternative, allowing for a more robust assessment of the accuracy of their effects, as there is a well-documented relationship in the literature regarding this type of exercise.  A recently conducted systematic review investigating the effect of physical exercise on depression revealed that dance exercises combined with selective serotonin reuptake inhibitors (SSRIs), walking, or running were the treatments most likely to be effective in treating depression. Furthermore, the results indicate that the effect of exercise is proportional to its intensity, with vigorous activities showing better responses. However, the findings did not allow for the precise establishment of a dose-response relationship due to variability in the study protocols analyzed, particularly regarding frequency, duration, and weekly volume of activity [6]. In this regard, conducting the proposed review with aerobic exercises may be a viable option for refining the results. |
| Furthermore, there are several inconsistencies, such as ignoring the fact that vigorous-intensity exercise is already recognized as involving anaerobic effort. Claiming that exercise is aerobic solely based on heart rate and perceived exertion is scientifically imprecise. Measurements of lactate or gas analysis are necessary. Even interval exercise can be aerobic as long as the work-rest ratio does not promote an imbalance in blood lactate levels. | We appreciate our comments and agree with you. After a detailed analysis of the text regarding the definition of aerobic exercises, the authors recognized the need to refine the presented conceptualization. In fact, the observation made by the reviewer was highly relevant. Considering the inconsistency pointed out, we revised the passage to emphasize the dynamic interaction between energy systems, avoiding the classification of an exercise as aerobics solely based on heart rate or perceived exertion.  Please see the changes.  “In this context, physical exercise emerges as a promising intervention for improving mental health. The focus of this review will be the study of exercises with an aerobic predominance, commonly known as 'aerobic exercise,' which are characterized by the primary use of the oxidative pathway for energy production. This type of exercise involves long-duration activities with varying intensity levels, ranging from low (walking), moderate (light jogging, cycling), to high (continuous running near VO₂max, competitive cycling, endurance swimming). The energy demand in these efforts is primarily sustained by mitochondrial metabolism [2].  However, the analysis of these exercises should not be based solely on perceived effort or heart rate, as all energy pathways operate simultaneously, varying according to the duration and intensity of the exercise [2]. Thus, efforts are categorized as follows: explosive efforts (up to 6 seconds), dominated by the phosphagen system (ATP-CP); high-intensity efforts (6 seconds to 1 minute), where anaerobic glycolysis predominates but with an increasing aerobic contribution; and prolonged endurance efforts (above 1–2 minutes), where oxidative phosphorylation plays a primary role, although anaerobic glycolysis still contributes during intensity transition phases [2]. |
| - Moreover, the grammar and verb tense used in the article suggest to the reader that something was discovered, but in reality, no results are presented. | Thank you. We have made multiple revisions regarding this request for grammatical review, and the adjustment of verb tense in the manuscript has been addressed. Additionally, we have highlighted it in red in the discussion section. We hope that the changes made are aligned with the requested modifications.  We also appreciate all the considerations and suggestions provided, which were of great importance for the improvement of this review and contributed significantly to the field of scientific knowledge.  The reference to Figure 1 has also been included in the text as requested. It is located on page 6 of the manuscript and highlighted in red font. |
