# Supplementary material for: Effectiveness of aerobic physical exercise on depression symptoms in adults: A protocol for developing a systematic review and meta-analysis of randomized clinical trials
Source: PLoS One. 2025 Jun 2;20(6):e0314846. doi: 10.1371/journal.pone.0314846 (PMC12129328; doi:10.1371/journal.pone.0314846)
Supplement: Attachment — Submitted filename: Response to Reviewers -Plos One (1).docx [file pone.0314846.s003.docx]

**Response to Reviewers**

**Manuscript number:** PONE-D-24-51714

**Title:** Effectiveness of aerobic physical exercise on depression symptoms in adults: A protocol for developing a systematic review and meta-analysis of randomized clinical trials

**Dear Reviewers,**

First and foremost, I would like to express my gratitude, on behalf of all the authors of the manuscript, to the esteemed reviewers for raising extremely important considerations that are highly relevant for improving the article.

It is important to note to the distinguished reviewers that the manuscript submitted to PLOS ONE is a robust protocol for conducting a systematic review, aimed at gathering scientific evidence on the effect of aerobic exercise in the treatment of depression in adults.

We have made all the requested adjustments, which have been incorporated into the manuscript itself, with the changes highlighted in red font.
These modifications are also listed below:

| **Reviewer #2** | **Authors' response:** |
| --- | --- |
| Reviewer: It seems like you're pointing out that the research was more of a plan or outline rather than an actual study, without concrete results. | We appreciate your contribution and agree with your position. Please review the changes that were made to some parts of the abstract and the introduction.  In the abstract/objective: “This article presents a protocol for conducting a systematic review with meta-analysis, aimed at investigating the effects of aerobic exercise on reducing depression symptoms in adults.”  In the abstract/methods: “Data extraction will be performed using specific forms, while the methodological quality of studies on physical exercise will be assessed with the TESTEX tool, and the risk of bias will be evaluated using the Cochrane RoB 2.0 method”.  In the abstract/conclusion: “It is expected that the systematic review following this protocol will identify the effective dose-response to reduce depression levels and provide an understanding of the mechanisms through which aerobic exercise influences depression”.  Some parts of the introduction:  “Although the benefits of aerobic exercise in improving depressive symptoms are well documented, the existing literature shows high variability in terms of modality, intensity, frequency, and duration of exercise. Furthermore, the underlying mechanisms of these effects are still not fully understood [3].“  “Recent reviews, including network meta-analyses, suggest that both the dose and the modality of exercise may influence clinical outcomes [8]. However, many existing systematic reviews and meta-analyses have methodological limitations that prevent definitive conclusions. In light of this, the isolated investigation of aerobic exercise using a robust and clearly defined methodology may contribute to a more accurate understanding of its effectiveness on depressive symptoms.”  “In this regard, the development of this protocol focused exclusively on aerobic exercises represents a viable strategy to guide a future systematic review and refine the results obtained in the scientific literature.  This protocol proposal aims to guide a future systematic review that will seek to answer the following question: what are the effects of aerobic exercise on reducing depression levels in adults? With this protocol, we intend to provide researchers and healthcare professionals with a rigorous and transparent methodological foundation for conducting a systematic review and meta-analysis of randomized clinical trials on the topic.” |
| **Reviewer #3** | |
| The article presents scientific merit and contributes to the advancement of scientific knowledge about an efficient roadmap (a strategy) for conducting systematic review studies and meta-analysis of randomized clinical trials. The authors describe the methodological procedures for conducting scientific studies of this nature using different types of aerobic physical exercise and their outcomes related to symptoms of depression in adults. The protocol outlined in the article can be applied in conducting this study design to minimize the risk of methodological errors. However, I suggest some adjustments to improve the article.  - I suggest changing the title of the article to: Effectiveness of aerobic physical exercise on depression symptoms in adults: A protocol for developing a systematic review and meta-analysis of randomized clinical trials | We appreciate your input and concur with your position. Kindly review the alterations that have been made.  “Effectiveness of aerobic physical exercise on depression symptoms in adults: A protocol for developing a systematic review and meta-analysis of randomized clinical trials” |
| Introduction: - The authors must make it clear that the aim of this study was to develop a protocol of methodological procedures to conduct systematic review studies with meta-analysis of randomized clinical trials on different types of aerobic physical exercises and their outcomes related to symptoms of depression in adults. | We appreciate your input and concur with your position. Kindly review the alterations that have been made.  “This protocol proposal aims to guide a future systematic review that will seek to answer the following question: what are the effects of aerobic exercise on reducing depression levels in adults? With this protocol, we intend to provide researchers and healthcare professionals with a rigorous and transparent methodological foundation for conducting a systematic review and meta-analysis of randomized clinical trials on the topic.” |
| -Methods: Authors must include the reference to the PICOS strategy (Methley AM, Campbell S, Chew-Graham C, McNally R, Cheraghi-Sohi S. PICO, PICOS and SPIDER: a comparison study of specificity and sensitivity in three search tools for qualitative systematic reviews. BMC Health Serv Res. 2014;14:579. doi:10.1186/s12913-014-0579-0).  - In this section, there are repeated passages about the PICOS strategy. I suggest leaving only one citation in the text. | We appreciate your input and concur with your position. Kindly review the alterations that have been made.  “Thus, the clinical PICOS question, widely recommended to guide systematic reviews [10], will be formulated as follows: Population (adults); Intervention (aerobic exercise); Comparator (other types of exercise/placebo/control); Outcomes (improvement of depressive symptoms); Study types (randomized controlled trials – RCTs).”  “**Eligibility criteria**  We will follow these criterias: Participants will include adults (>18 years), excluding individuals with chronic degenerative diseases or severe psychiatric disorders. The intervention will focus on aerobic exercise (e.g., walking, running), with comparators including other interventions or control groups. The primary outcome will be depressive symptoms, while training volume and frequency will be considered secondary outcomes. Only studies utilizing validated depression assessment scales will be included, such as the Beck Depression Inventory (BDI), Hamilton Depression Rating Scale (HAM-D), Patient Health Questionnaire (PHQ-9), Center for Epidemiologic Studies Depression Scale (CES-D), Montgomery-Åsberg Depression Rating Scale (MADRS), Zung Self-Rating Depression Scale (Zung SDS), Geriatric Depression Scale (GDS), and Hospital Anxiety and Depression Scale (HADS), among others. Eligible study types will be randomized clinical trials (RCTs), no restriction of language, while ongoing clinical trials with preliminary results will be excluded. Additionally, studies involving populations with non-communicable chronic diseases (e.g., hypertension, diabetes), case reports, preprints, narrative and systematic reviews, and observational studies will not be considered for inclusion.” |
| - Authors can indicate the TESTEX tool (Smart NA, Waldron M, Ismail H, et al. Validation of a new tool for the assessment of study quality and reporting in exercise training studies: TESTEX. Int J Evid Based Healthcare. 2015;13(1):9–18. doi:10. 1097/XEB.0000000000000020), because it is more suitable for assessing the methodological quality of studies involving physical exercise. While RoB2 will be used to assess the risk of bias. | We appreciate your input and concur with your position. Kindly review the alterations that have been made.  “The methodological quality assessment of the included studies will be carried out by two independent reviewers (L.N.S; M.I.K) using the TESTEX tool (Tool for the assEssment of Study qualiTy and reporting in EXercise), which was specifically developed for clinical trials involving physical exercise interventions. This scale, validated by Smart et al. [12], includes criteria addressing both methodological quality and reporting quality of the studies, with a total possible score of 15 points—5 for study quality and 10 for reporting quality. TESTEX stands out for addressing relevant and specific aspects of exercise interventions, such as adherence to the protocol, exercise intensity, and volume, making it more appropriate than other tools for use in exercise-related reviews.  In addition, the Cochrane Risk of Bias 2.0 tool (RoB 2.0) will be used to assess the risk of bias in the included studies [13]. This tool evaluates the following domains: the randomization process, deviations from the intended intervention, missing outcome data, measurement of outcomes, selection of the reported results, and the overall judgment of the risk of bias.” |
| - Authors must indicate the I2 values to use fixed or random effects in the decision-making process in the meta-analysis (Forest-Plot). | We appreciate your input and concur with your position. Kindly review the alterations that have been made.  “The meta-analysis will be conducted using fixed-effect or random-effects models, depending on the heterogeneity among the included studies. The degree of heterogeneity will be assessed using the I² statistic. A fixed-effect model will be applied when heterogeneity is low (I² ≤ 50%), while a random-effects model will be used when heterogeneity is moderate or high (I² > 50%) [15].” |
| - Authors must indicate the classification and application of the categories in the GRADE tool. | We appreciate your input and concur with your position. Kindly review the alterations that have been made.  “In this protocol, the GRADE approach will be applied to assess the certainty of the evidence for each outcome included in the systematic review and meta-analysis. The evaluation will be based on the following domains: risk of bias, inconsistency of results, indirectness of evidence, imprecision of estimates, and potential publication bias. Two reviewers will independently assess each domain, and disagreements will be resolved by consensus or by a third reviewer. The results will be summarized in a GRADE evidence profile and presented in a Summary of Findings table. GRADE categorizes both the level of evidence and the strength of recommendations into four categories: high, moderate, low, and very low [14].” |
| - Authors must indicate when a funnel plot should be used (minimum of 10 articles). | We appreciate your input and concur with your position. Kindly review the alterations that have been made.  “The presence of bias in relation to estimates against sample size will be analyzed using a funnel plot, which will be generated only if at least 10 studies are included in the meta-analysis, as recommended to ensure reliability of asymmetry assessments [15].” |
| - Authors must indicate the main points of analysis and interpretation of a forest plot. | We appreciate your input and concur with your position. Kindly review the alterations that have been made.  “We will analyze a forest plot by considering the effect size and 95% confidence intervals (CIs), heterogeneity (I²), study weight, and the pooled effect size. All of these points should be interpreted in relation to the null value, and potential publication bias should be evaluated using a funnel plot or Egger’s test.” |
| Discussion: In the discussion section, authors must highlight in depth the main factors that can generate high heterogeneity (I2) in the meta-analysis and how this can be considered as one of the limitations of a systematic review study with meta-analysis. | We appreciate your input and concur with your position. Kindly review the alterations that have been made.  “The systematic review studies conducted so far, although following rigorous methodologies and providing relevant contributions to the scientific field, present some methodological limitations. Anticipated limitations and challenges include the following issues: the use of small sample sizes, lack of clarity regarding the specific mechanisms through which exercise contributes to the improvement of depression, and variations in the intensity protocols adopted. In addition, one of the main methodological challenges expected in the conduction of this review is the high heterogeneity among the studies to be included. Differences may arise in exercise modalities, levels of depressive symptoms, different intensities, frequency and duration of exercise, presence of other comorbidities, supervised versus unsupervised exercise, individual versus group exercise, and gender differences, as men and women may respond differently. However, this review aims precisely to highlight these issues and deepen the understanding of their implications.” |
