# Supplementary material for: Effectiveness of aerobic physical exercise on depression symptoms in adults: A protocol for developing a systematic review and meta-analysis of randomized clinical trials
Source: PLoS One. 2025 Jun 2;20(6):e0314846. doi: 10.1371/journal.pone.0314846 (PMC12129328; doi:10.1371/journal.pone.0314846)
Supplement: Attachment — Submitted filename: Response to Reviewers -Plos One (2).docx [file pone.0314846.s004.docx]

**Response to Reviewers**

**Manuscript number:** PONE-D-24-51714

**Title:** Effectiveness of aerobic physical exercise on depression symptoms in adults: A protocol for developing a systematic review and meta-analysis of randomized clinical trials

**Dear Reviewers,**

First and foremost, I would like to express my gratitude, on behalf of all the authors of the manuscript, to the esteemed reviewers for raising extremely important considerations that are highly relevant for improving the article.

It is important to note to the distinguished reviewers that the manuscript submitted to PLOS ONE is a robust protocol for conducting a systematic review, aimed at gathering scientific evidence on the effect of aerobic exercise in the treatment of depression in adults.

We have made all the requested adjustments, which have been incorporated into the manuscript itself, with the changes highlighted in red font.
These modifications are also listed below:

| **Reviewer #4** | **Authors' response:** |
| --- | --- |
| Reviewer: Many thanks for the invitation to review this manuscript. In this paper the authors present a protocol for a systematic review and meta-analysis of randomized clinical trials on the effectiveness of aerobic physical exercise on depression symptoms in adults. The study has good elements of originality. Below are few suggested minor comments to address. Introduction - Please incorporate literature on the impact of depression and draw these together to urgency for effective strategies  Methods - Participants: It is not clear whether participants would need to have depression or not. Please indicate this**.** | We appreciate your input and concur with your position. Kindly review the alterations that have been made.  “Since it is a condition often aggravated by other diseases, such as substance use disorders, diabetes, and heart diseases, which not only increase the risk of developing depression but are also affected by it, establishing a cycle that further compromises the health and quality of life of those affected [2].  In general, mental health problems, neurological disorders, and substance use disorders account for 13% of the global burden of disease, with depression alone responsible for 4.3% of this total, women are the most affected [3]. In 2019, there were 290.2 million cases of depression worldwide, an increase of 59.28% compared to 1990 [3].  Projections based on the Bayesian age-period-cohort (BAPC) model indicate that, by 2030, global age-standardized rates are expected to remain stable. However, the absolute number of depression cases is likely to increase, potentially reaching 108.9 million among men and 164.9 million among women [4].  The research conducted by Liu et al. [5] reinforces these projections by indicating stability in age-specific incidence rates between 1990 and 2017, along with an increase in the total number of global depression cases, reaching 258 million in 2017, a growth of 49.86% compared to 1990. Regions with low Socio-Demographic Index (SDI) face the greatest impacts, mainly due to the lack of investment in mental health. In these areas, depression often remains undiagnosed or inadequately treated, resulting in higher morbidity and disability.  In the United States, the annual prevalence of depression among adults reached 8.9 million people undergoing pharmacological treatment, with approximately 30.9% (2.8 million) being treatment-resistant [6].  Treatment-resistant depression is defined as the failure of at least two adequate trials with different antidepressants, over a minimum period of four to six weeks, at the maximum recommended dose [7,8].  Given the multifaceted nature of the disorder, significant gaps are still observed in the studies, attributed in part to the diversity of symptoms and the limitations of current technologies to investigate the human brain in real time, both at the circuit and synapse levels [9]. The condition can be triggered by biological changes [10], as well as by psychosocial influences [11,12].  In view of the aforementioned issues, researchers and scholars in the field have been seeking effective strategies capable of responding, in an individualized manner, to the complexities of depressive disorder, considering its multifactorial nature. In this context, physical exercise has emerged as a promising intervention for mental health, especially among individuals with antidepressant-resistant depression. Furthermore, evidence indicates that even in these cases, physical exercise, when used as an adjunct to pharmacological treatment, demonstrates significant potential for reducing depressive symptoms, including non-remissive cases, as well as improving quality of life, sleep, and vitality [13]”.  - In the Research Question section, we added 'adults with depression' to the population.  “Thus, the clinical PICOS question, widely recommended to guide systematic reviews [22], will be formulated as follows: Population (adults with depression); Intervention (aerobic exercise); Comparator (other types of exercise/placebo/control); Outcomes (improvement of depressive symptoms); Study types (randomized controlled trials – RCTs).”  - In the Eligibility Criteria section, we also added the indication at the beginning of the paragraph.  “Participants will include adults (>18 years) diagnosed with depression, excluding individuals with chronic degenerative diseases or other associated severe psychiatric disorders.” |
